# Impact of nanosilver on various DNA lesions and *HPRT* gene mutations – effects of charge and surface coating

**DOI:** 10.1186/s12989-015-0100-x

**Published:** 2015-07-24

**Authors:** Anna Huk, Emilia Izak-Nau, Naouale el Yamani, Hilde Uggerud, Marit Vadset, Beata Zasonska, Albert Duschl, Maria Dusinska

**Affiliations:** Health Effects Laboratory, MILK, NILU, Kjeller, Norway; Department of Molecular Biology, University of Salzburg, Salzburg, Austria; Bayer Technology Services GmbH, Leverkusen, Germany; Inorganic Group, MILK, NILU, Kjeller, Norway; Institute of Macromolecular Chemistry, Academy of Sciences of the Czech Republic, Prague, Czech Republic

**Keywords:** Silver nanomaterials, Surface charge, Surface coating, Uptake and localization, Cytotoxicity, DNA damage, Genotoxicity/mutagenicity

## Abstract

**Background:**

The main goal of this research was to study the interactions of a fully characterized set of silver nanomaterials (Ag ENMs) with cells *in vitro*, according to the standards of Good Laboratory Practices (GLP), to assure the quality of nanotoxicology research. We were interested in whether Ag ENMs synthesized by the same method, with the same size distribution, shape and specific surface area, but with different charges and surface compositions could give different biological responses.

**Methods:**

A range of methods and toxicity endpoints were applied to study the impacts of interaction of the Ag ENMs with TK6 cells. As tests of viability, relative growth activity and trypan blue exclusion were applied. Genotoxicity was evaluated by the alkaline comet assay for detection of strand breaks and oxidized purines. The mutagenic potential of Ag ENMs was investigated with the *in vitro HPRT* gene mutation test on V79-4 cells according to the OECD protocol. Ag ENM agglomeration, dissolution as well as uptake and distribution within the cells were investigated as crucial aspects of Ag ENM toxicity. Ag ENM stabilizers were included in addition to positive and negative controls.

**Results:**

Different cytotoxic effects were observed including membrane damage, cell cycle arrest and cell death. Ag ENMs also induced various kinds of DNA damage including strand breaks and DNA oxidation, and caused gene mutation. We found that positive Ag ENMs had greater impact on cyto- and genotoxicity than did Ag ENMs with neutral or negative charge, assumed to be related to their greater uptake into cells and to their presence in the nucleus and mitochondria, implying that Ag ENMs might induce toxicity by both direct and indirect mechanisms.

**Conclusion:**

We showed that Ag ENMs could be cytotoxic, genotoxic and mutagenic. Our experiments with the *HPRT* gene mutation assay demonstrated that surface chemical composition plays a significant role in Ag ENM toxicity.

## Background

Silver in nano-form, called also engineered silver nanomaterials (Ag ENMs) or simply nanosilver, is of size less than 100 nm, mostly at oxidation stage Ag^0^ with presence of Ag ions [1, 2]. Due to high reactivity, high temperature stability and low volatility, low cost of production and antibacterial properties, Ag ENMs are one of the most often used ENMs, and are present in a wide range of products [3, 4]. Ag ENMs have applications in water purification filters, textiles, catalysts, food packages and cosmetics [5–8]. Nevertheless, the most profitable application of Ag ENMs is in medical products such as drug delivery, diagnostic tools, bone cement, coatings for orthopedic stockings and implants, and pharmaceuticals for dermatitis, acne and ulcerative colitis treatment [9–12]. Increasing numbers of products based on Ag ENMs demand relevant *in vitro* toxicology research on those materials, with special attention given to correlate physical properties of Ag ENMs with harmful effects [13].

Intensive investigation of ENM toxicity in the last decade has brought many inconclusive and controversial results. A number of studies have reported cytotoxic effects of Ag ENMs, such as inhibition of cell proliferation, cell membrane damage, apoptosis and necrosis [14–19]. It was also found that Ag ENMs can interact with DNA, inducing different DNA lesions such as strand breaks, DNA oxidation and DNA adducts [15, 18–21]. In nanotoxicology research it is fundamentally important to understand the link between physico-chemical properties of ENMs and their toxicity, because even small changes in ENM structure can affect final biological responses [13, 22]. Ag ENMs are not uniform compounds but materials with different sizes, shapes, and with different surface charge, composition and functionalization. Previous toxicology evaluations of Ag ENMs were mostly focused on size-related toxicity [23–27] demonstrating significant impact of size on biological response. However, some studies suggest that not size but surface charge can play a crucial role in the mode of action of Ag ENMs [28, 29]. Suresch *et al.* [28] and el Badawy *et al.* [29] demonstrated that the cationic Ag ENMs are more toxic for both mammalian and bacterial cells. However, the correlation between surface charge and toxicity of Ag ENMs is not straightforward. Due to the fact that only one cationic Ag ENM has been tested in cited studies, it cannot be definitely proved that observed effects are only related to surface charge and not to surface chemical composition.

Therefore, to better understand the mechanism of Ag ENMs toxicity, in this study we focused most on effects of Ag ENM surface charge and surface composition on cell toxicity.

We tested six different Ag ENMs, two for each surface charge, from the same sources, synthesized by the same method and fully characterized by standard techniques. Two different stabilizers per charge were selected to distinguish between effects of surface charge and surface chemical composition. Trisodium citrate and sodium dodecyl sulphate (SDS) were selected to ensure a negative charge on Ag ENMs, BYK9067® and chitosan for a positive charge and Tween® 80 and Disperbyk 192® for a neutral charge. For the toxicity study, a range of different endpoints was addressed and standard methods have been applied.

In the present study we used the human B-lymphoblastoid (TK6) cell line, and circulating blood cells. As a representative cell model for nanotoxicology studies, TK6 cells were validated in a previous study against human peripheral blood cells and they were found to be a relevant model for blood cells in nanotoxicology studies [30]. Additionally, to study mutations induced by ENMs, we used Chinese hamster lung fibroblast cells (V79-4) according to the test guideline OECD 476, as a continuation of our previous experiments on size-dependent mutagenicity of Ag ENMs [25].

## Materials and methods

### Ag nanomaterials

Ag ENMs with the same size, shape and specific surface area but with different charges and surface compositions were synthesized by chemical reduction of silver nitrate (AgNO_3_; Heraeus, Germany) using sodium borohydrate (NaBH_4_; ACROS Organics, Germany) (modified method of Creighton *et al.,* [31]). A variety of coupling agents were used to stabilize ENMs from agglomeration: 3-sodium citrate (Na_3_C_6_H_5_O_7_; Fisher Scientific, Germany) and sodium dodecyl sulfate (SDS; Sigma-Aldrich, Germany) - negatively charged; chitosan (Sigma, Germany) and BYK-9076® (BYK-Chemie, Germany) - positively charged; Tween 80® (Sigma-Aldrich, Germany) and Disperbyk-192® (BYK-Chemie, Germany) - neutral.

The investigated Ag ENMs were characterized by a combination of different techniques (Table 1). The average size/size distribution of primary Ag ENMs was determined by transmission electron microscopy (TEM; Phillips CM20, 200 keV) and dynamic light scattering (DLS; 90Plus, Brookhaven Instruments Corporation). TEM was additionally applied to define the Ag ENM shape. For TEM analysis, the stock dispersions were pipetted onto cobalt grids covered with polyvinyl formal/carbon (S162, Plano GmbH) and left to evaporate. A series of 10 images were selected to estimate the ENM size/size distribution using the analySiS pro software (Olympus). DLS measurements were performed in 10 mm polystyrene cuvettes at 25 °C using a He-Ne laser (673 nm). The ZetaPALS Particle Sizing Software ver. 4.10 was used to calculate the ENM size. The results are given as Z-average values (±SD) of the number, volume and intensity size distributions. The zeta potential was determined with the same equipment using phase analysis light scattering Zeta Potential Analyzer ver. 3.29. The DLS and zeta potential measurements were performed five times per batch of ENMs. The crystallite size and crystalline phase were evaluated by X-ray diffraction (XRD; PANalytical EMPYREAN PIXcel) at a voltage of 40 kV and a current of 40 mA with Cu Kα and Kβ radiation. The stock dispersions were dried onto a silicon surface. The chemical and elemental composition of the ENMs were examined by X-ray photoelectron spectroscopy (XPS; PHI VersaProbe 5000, MultiPack ver. 9.2 software) equipped with a monochromated Al Kα X-ray beam scanned over an area of 600 μm × 400 μm or 1400 μm × 100 μm at a fixed take-off angle of 45°. The stock dispersions were dried onto an indium surface.

**Table 1:** Physical and chemical characterization of Ag ENMs

		Anionic Ag ENMs		Neutral Ag ENMs		Cationic Ag ENMs	
Name		Ag_Citrate	Ag_SDS	Ag_Disperbyk	Ag_Tween	Ag_Byk	Ag_Chitosan
Stabilizer		Sodium citrate 0.025 %	SDS 0.05 %	Disperbyk 192® 0.05 %	Tween 80® 0.1 %	BYK 9076® 0.025 % in 0.05 % acetic acid	Chitosan 0.05 % in 0.1 % acetic acid
Surface charge [mV]^a^	In solvent	- 42.26 ± 1.64	−33.23 ± 0.90	- 3.02 ± 4.12	−1.13 ± 1.73	+26.56 ± 1.67	+52.40 ± 1.79
	In RPMI 1600 Medium	−8.43 ± 0.84	−9.8 ± 1 .56	−3,38 ± 1.27	−1.2 ± 1.36	−9.8 ± 0.89	−4,83 ± 0.3
Surface chemistry [Atom %]		C 44.6; O 41.6; Na 9.2; N 3.0; Ag 1.7	C 48.1; O 40.2; Na 5.9; Ag 3.6; N 2.0; S 0.2	C 72.8; O 24.5; Ag 1.6; N 1.0	C 71.5; O 25.7; Na 2.6; Ag 0.2	C 69.5; O 16.3; N 9.4; Ag 4.7	C 46.3; O 41.3; N 7.3; Na 3.4; Ag 1.7
Size/size distribution & aggregation/agglomeration state [nm]^a^	In solvent	DLS: 5.4	DLS: 5.0	DLS: 9.1	DLS: 8.0	DLS: 9.2	DLS: −
		TEM: 5.9 ± 2.3	TEM: 6.2 ± 2.9	TEM: 6.9 ± 2.8	TEM: 6.1 ± 2.1	TEM: 10.5 ± 2.5	TEM: 9.8 ± 2.1
		XRD: 6.3	XRD: 6.8	XRD: 5.9	XRD: 6.3	XRD: 6.8	XRD: 7.1
	In RPMI 1600 medium	DLS:	DLS:	DLS:	DLS:	DLS:	DLS:
		P1: 133 ± 67.56	P1: 137.4 ± 78.24	P1: 391.5 ± 10.9	P1: 49.73 ± 10.9	P1: 34.83 ± 2.54	P1: 763.7 ± 379.4
		P2 : 22.04 ± 9.2	P2: 17.14 ± 5.31	P2: 41.18 ± 10.3			P2: 127.4 ± 37.93
Shape		spherical	spherical	spherical	spherical	spherical	spherical
Crystal structure		cubic	Cubic	cubic	cubic	cubic	cubic
Concentration [μg/ml]		100	100	100	100	100	100

^a^expressed as mean ± SD of 3 independent replicates

In addition, stability of Ag ENMs in cell culture medium (RPMI 1600, 10 % FBS) was investigated with Zetasizer Nano-ZS Model ZEN3600 (Malvern Instruments; Malvern, UK). Ag ENM stability was measured in 1.5 ml disposable cuvettes (Kartell, Italy) and 0.5 ml disposable folded capillary cells (for zeta potential measurements, Malvern, UK) at a controlled temperature of 37 °C after 24 h incubation in cell culture medium.

### Cultivation of cells

TK6 cells were obtained from the European Collection of Cell Culture (ECACC, Cat. 95111735). Cells were cultivated in RPMI 1640 culture medium (Sigma) supplemented with 10 % heat-inactivated fetal bovine serum (FBS, 20 min, 55 °C), 100 U/ml penicillin and 100 μg/ml streptomycin in a humidified atmosphere (5 % CO_2_ and 37 °C). 

Chinese hamster lung fibroblasts (V79-4) obtained from the European Collection of Cell Culture (ECACC, Cat.93010723) were cultured in flasks in DMEM low glucose medium (Sigma), with activated 10 % FBS, 100 U/ml penicillin and 100 μg/ml streptomycin and 200 mM L-glutamine in a humidified atmosphere (5 % CO_2_ and 37 °C). Both cell lines were tested for Mycoplasma with the MycoAlert™ PLUS Mycoplasma Detection Kit, before use. This kit detects the activity of mycoplasmal enzymes (detects all the main mycoplasma contaminants). Tests were performed after the first passage and no mycoplasma contamination was detected.

### Treatment of cells

TK6 cells were seeded in 24-well plates to evaluate the relative growth activity RGA, for TBE assay and for morphology observation, as well as in 96-well plates for the CA. To prepare cells for TEM, the cells were seeded in 100 mm Petri dishes at the density required. Volumes of exposure solutions and numbers of cells were adjusted to give equal Ag ENM concentrations in all assays. Ag ENM concentrations were expressed in mass units per ml (μg/ml), per exposure surface (μg/cm^2^), per cell (μg/cell); or in surface area of ENMs per ml (calculated on the basis of primary ENM sizes), per exposure surface, per cell (cm^2^/ml, cm^2^/cm^2^, cm^2^/cell). The information is summarized in Table 2.A. ENM mass per cell units of concentration was calculated in the following approach: Units [ENM mass/cell] = (Ag ENM concentration [μg/ml] * volume)/number of exposed cells. V79-4 cell number was counted immediately before the exposure from an additional plate which was seeded in the same manner and number as for the experiment. TK6 cells were counted (by Automatic cell counter) immediately before the experiment.

**Table 2:** Concentrations of Ag ENMs (A) and Ag ENM stabilizers (B) applied for different assay. Ag ENM concentrations are expressed in mass units [per volume/per exposure area/per cell] and in area of ENMs [per volume/per exposure area/per cell]. Stabilizer concentrations are expressed in % w/v

	A: Ag ENMs concentrations					
	Mass units			Surface area of ENMs		
	[μg/ml]	[μg/cm^2^]	[pg/cell]	[cm^2^/ml]	[cm^2^/cm^2^]	[cm^2^/cell]
CA RGA TBE CM	1	0.31	˜6.67	0.72	0.22	˜ 4.79×10^−6^
	2	0.63	˜ 13.33	1.44	0.45	˜ 9.59×10^−6^
	4	1.25	˜ 26.67	2.88	0.9	˜ 1.92×10^−5^
	8	2.5	˜ 53.33	5.75	1.8	˜ 3.87×10^−5^
	10	3.13	˜ 66.67	7.19	2.25	˜ 4.84×10^−5^
GMA	2	0.63	˜ 19.73	1.44	0.45	˜ 1.42×10^−5^
	4	1.25	˜ 39.47	2.88	0.9	˜ 2.84×10^−5^
	8	2.5	˜ 78.93	5.75	1.8	˜ 5.68×10^−5^
B: Ag ENM stabilizers concentrations						
	Citrate	SDS	Disperbyk	Tween	Byk	Chitosan
TBE	0.0025	0.005	0.005	0.01	0.0025	0.005
RGA	0.0025	0.005	0.005	0.01	0.0025	0.005
CM	0.002	0.005	0.005	0.01	0.0025	0.005
CA	0.002	0.004	0.004	0.008	0.002	0.004
GMA	0.002	0.004	0.004	0.008	0.001	0.002

*TBE* Trypan Blue exclusion assay, *RGA* Relative Growth activity, *CM* cells morphology, *CA* Comet assay, *GMA* Gene mutation assay

Additional TK6 cells were treated with only the Ag ENM stabilizer at a concentration equal to the stabilizer concentration used in the highest tested Ag ENM samples. Data are summarized in Table 2.B.

### Uptake and cellular localization

TK6 cells were exposed to Ag ENMs (2.5 μg/cm^2^: Ag Citrate, Ag_SDS, Ag_Disperbyk and Ag_Tween and 1.5 μg/cm^2^: Ag_Chitosan and Ag_Byk) in 100 mm Petri dishes (7.8 × 10^6^ cells/Petri dish). After 24 h cells were fixed in 2.5 % glutaraldehyde in 0.1 M Sorensen phosphate buffer (pH 7.3) and left overnight. Next day, cells were washed with 0.1 M Sorensen phosphate buffer (pH 7.3) and post-fixed in 1 % osmium tetroxide in deionized water. Samples were dehydrated in increasing concentrations of ethanol (from 10 to 100 %, 10 min each step, centrifuged every time at 200 g for 5 min), immersed in ethanol/Epon (1:1 v/v) mixture and embedded in pure Epon (2 h at 37 °C and polymerised for 24 h at 60 °C). Sections (~80 nm) were cut using a diamond knife on an ultra-microtome (Leica EM UC6) and mounted on copper grids. From each sample of exposed cells 5 grids with cell sections (approximate 20–40 cells per section) were prepared. Before image acquisition, sections were stained using uranyl acetate and lead citrate. All images were acquired on an FEI TECNAI 120 TEM (120 kV). Under investigation only the cells without damaged membrane (not in necrosis or advanced apoptosis) were taken. For each Ag ENM we tried to access minimum 20 images of individual ENMs. Experiments were run in triplicate.

### Trypan blue exclusion assay (TBE)

To test for cell membrane integrity (assumed to be a measure of cell viability), TK6 cells (4.4 × 10^5^ cells/well) were exposed to Ag ENMs for 2 and 24 h at a range of concentrations (0.31, 0.63, 1.25, 2.5, 3.13 μg/cm^2^) in 6-well plates. After exposure, TK6 cells were disaggregated in the medium. About 10 ml of cell suspension was mixed with 10 ml trypan blue (0.4 %, Invitrogen) and viability (percentage of trypan blue positive cells) was measured using a Countess Automated Cell Counter (Invitrogen). Cell viability was determined according to the formula:

$$ TBE\left(\%\right)=\left(1-\frac{number\  of\  steined\  cells\ }{number\  of\  total\  cells}\right)\times 100\% $$

Six stabilizers were used as controls according to Table 2.B. Interference of Ag ENMs with Countess™ Automated Cell counter was studied in a cell-free system.

### Relative growth activity (RGA)

To assess cell proliferation as well as cell death, TK6 cells (4.4 × 10^5^ cells/well) were exposed to Ag ENMs for 24 h at a range of concentrations (0.31, 0.63, 1.25, 2.5, 3.13 μg/cm^2^) in 6-well plates. After 24 h exposure cells were disaggregated and counted using a Countess™ Automated Cell Counter (Invitrogen). RGA was calculated according to the formula:

$$ RGA\;\left(\%\right)=\frac{\left( number\  of\  cells\  at\  day\ 2/ number\  of\  seeded\  cells\  at\  day\ 0\right) in\  exposed\  cultures\ }{\left( number\  of\  cells\  at\  day\ 2/ number\  of\  seeded\  cells\  at\  day\ 0\right) in\  unexposed\  cultures}\times 100\% $$

Six stabilizers were used as controls according to Table 2.B.

### Cell morphology

To observe changes in cell morphology, TK6 cells (4.4×10^5^ cell/well) were treated with Ag ENMs (3.13 μg/cm^2^) in 6-well plates for 2 and 24 h and observed under an optical microscope (Leica, model DM-IL). Images were captured under 100× magnification (Motic, model Motican 3 software Motic Images 2.0 ML). Cell morphology was analyzed visually by comparing about 300 images of untreated and treated cells. Six stabilizers were used as control according to Table 2.B.

### Comet assay (CA)

Single-cell gel electrophoresis, the CA, is a sensitive and relatively simple method to study specific DNA lesions such as single and double strand breaks and DNA oxidation. Due to the high number of samples to be tested, we developed a very efficient semi-high-throughput method to analyze the genotoxic effect of Ag ENMs [32].

TK6 cells (1.5 × 10^4^ cells /well) were exposed in 96-well plates for 2 or 24 h at a range of concentrations (0.31, 0.63, 1.25, 2.5 μg/cm^2^). After exposure, 15–40 μl of cell suspension were transferred to a 96 well U-bottom plate, and mixed with 200 μl of LMP agarose (0.8 % in PBS). 10 μl of this mixture was dropped on glass slides (pre-coated with 0.5 % standard agarose) - 2 drops per concentration, 12 drops per slide. Slides were placed in cold lysis solution (2.5 M NaCl, 0.1 M EDTA, 10 mM Tris, 10 % Triton X-100, pH 10). After overnight lysis, slides were submerged in alkaline solution (0.3 M NaOH, 1 mM EDTA) for DNA unwinding for 20 min, followed by electrophoresis at 1.25 V/cm for 20 min in a standard CA electrophoresis tank. Slides were then washed in PBS followed by cold water and allowed to dry overnight. Slides were stained with SybrGold (0.1 l/ml in TE buffer - 10 mM TrisHCl, 1 mM Na_2_EDTA, pH 7.5–8, Invitrogen), covered with a cover slip and examined with a fluorescence microscope (Leica DMI 6000 B). Images of comets were scored using image analysis Comet Assay IV software (Perceptive Instruments), calculating mean % DNA in tail from 50 comets per gel.

For detection of DNA base oxidation we used the modified CA with the bacterial repair enzyme formamidopyrimidine DNA glycosylase (FPG, provided by Professor Andrew Collins, University of Oslo, Norway) [33]. Just after lysis, slides were washed with FPG buffer (no enzyme) (40 mM HEPES, 0.1 M KCl, 0.5 mM EDTA, 0.2 mg/ml bovine serum albumin, pH 8.0), placed in a special plate for enzyme incubation (Huk, NILU) and 30 μl of FPG in this buffer was added to each gel. Slides were covered with polypropylene foil and incubated for 30 min at 37 °C. Further steps were according to the standard CA protocol described above. Net FPG-sensitive sides (Net FPG) were calculated as the difference in % DNA in tail between samples with FPG incubation and samples with buffer incubation. As positive control for strand breaks (SBs), cells were treated with hydrogen peroxide (Sigma, 50 μM, 5 min on ice). As positive control for FPG-sensitive sites, cells were treated with the photosensitiser Ro19-8022 (Hoffman La Roche) plus visible light (1 μM in PBS, 5 min, on ice). Six stabilizers were used as controls according to Table 2.B.

### Mammalian *in vitro HPRT* gene mutation test, OECD 476

V-79-4 cells were plated on 6-well plates (1 × 10^5^ cells per well) and incubated at 37 °C. After 24 h, the cells were exposed to Ag ENMs for 24 h at a range of concentrations (0.63, 1.25, 2.5 μg/cm^2^). After exposure, medium was removed; cells were washed with PBS, trypsinized and re-suspended in 2 ml medium. 10^6^ cells were seeded in 100 mm Petri dishes (3.5 × 10^5^ cells/Petri dish, 3 dishes per sample to achieve 10^6^ cells per sample), grown in culture medium for 8 days, and split at days 3 and 5. Cells were harvested for mutant analysis at day 6 and 8 after treatment: cells were inoculated in 100 mm Petri dishes (2 × 10^5^ cells/Petri dish, 5 dishes per sample to achieve 10^6^ cells per sample). Cells were grown in selective medium containing 6-thioguanine (final concentration 5 μg/ml) for 10 days to form colonies. Mutant (6-thioguanine-resistant) colonies were stained with 1 % methylene blue and counted manually. Only colonies comprising at least 50 cells were counted.

For measurement of cell survival (plating efficiency, PE_0_ PE_6_ PE_8_) on days 0, 6 and 8 after exposure 100 cells were plated into 6-well plates (100 cells per well, 1 plate for each sample) and incubated for 7 days. Cells were then stained with 1 % methylene blue and counted manually. Thus survival was determined at the time of each mutation harvest and calculated on the basis of the number of colonies versus the number of inoculated cells.

Mutant frequency was calculated according to the following formula:

$$ Mutant\  frequency\left(x10\hat{\mkern6mu} 6\right)=\frac{number\  of\  mutant\  colonies}{number\  of\  surviving\  inoculated\  cells}\times 100 $$

PE was calculated according to the following formula:

$$ PE\left(\%\right)=\frac{number\  of\  colonies\  in\  exposed\  cultures}{number\  of\  colonies\  in\  unexposed\  cultures}\times 100\% $$

Cells treated with methyl methanesulfonate (MMS; 0.03 mM; 30 min, Sigma) were used as positive control. Six stabilizers were used as controls according to Table 2.B.

### Release of Ag ions from Ag ENMs in a biological medium

Concentrations of Ag ions in Ag ENM samples were evaluated by inductively coupled plasma mass spectrometry (ICP-MS) and expressed as % of total Ag in Ag ENMs stocks. Ag ions were isolated from Ag ENMs by (a) extraction (Izak-Nau E, Huk A, Reidy B, Uggerud H, Vadset M, Eiden S, Voetz M, Duschl A, Dušinska M, Lynch I: Impact of Storage Conditions and Storage Time on Silver Nanoparticle Physicochemical Properties and Implications for Biological Effects. Manuscript in preparation) and (b) by ultracentrifugation [s = 200 000 g, t = 1 h].

In addition, ICP-MS was applied to quantify release of Ag ions after incubation of Ag ENMs in RPMI culture medium for 24 h in a CO_2_ incubator at 37 °C. Samples were then ultracentrifuged (200 000 g) for 1 h. Supernatants were collected, mineralized with concentrated nitric acid (nitric acid ultrapure, Sigma) and analyzed by ICP-MS (Agilent Technologies 7700x Series).

### Statistical analysis

Data are expressed as mean ± SD of 3 independent experiments. The differences between the untreated controls and the treatment groups were calculated by one-way analysis of variance (ANOVA) and posttests were analyzed using Tukey’s test, to evaluate significant levels. Graph Pad Prism software, Microsoft® Excel and Daniels XL toolbox were used.

## Results

### Characterization of Ag ENMs

**Fig. 1:**
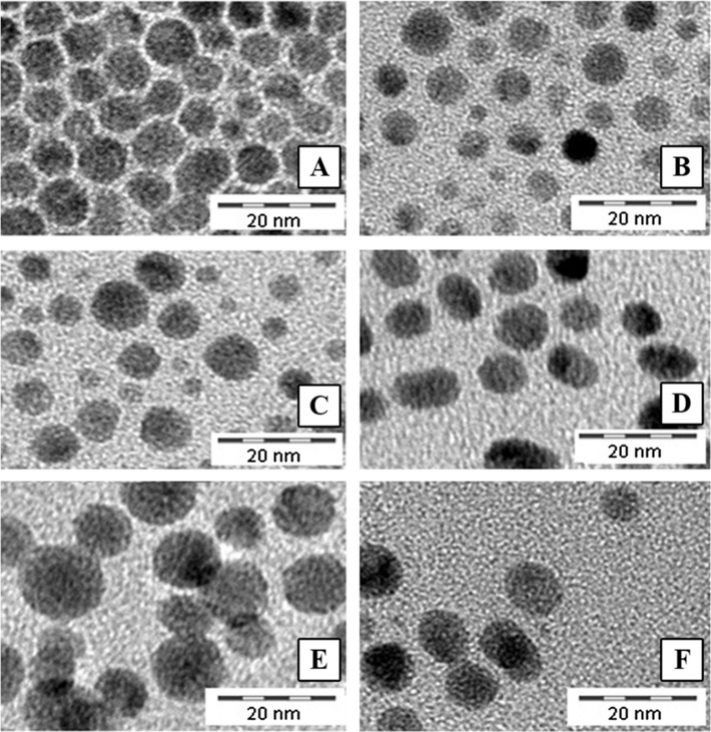
TEM characterization of pristine Ag ENMs: (**a**) Ag_Citrate, (**b**) Ag_SDS, (**c**) Ag_Disperbyk, (**d**) Ag_Tween, (**e**) Ag_Chitosan, (**f**) Ag_Byk

The Ag ENM characteristics are summarized in Table 1. DLS, TEM and XRD data indicated the ENM size to be in the region of 5–10 nm. In the case of DLS, the size analysis of Ag ENMs stabilized by chitosan was not possible to perform in solvent (the size exceeds 1 μm). Since the size of those Ag ENMs was confirmed by TEM and XRD (TEM: 9.8 ± 2.1 nm, XRD: 7.1 nm), it can be concluded that the big molecules of chitosan (which are normally not detected by TEM and XRD) disturbed the DLS analysis. TEM images also showed a quasi-spherical shape and monodispersal of the ENMs (Fig. 1). The characterization of Ag ENMs in cell culture medium (RPMI 1600; 10 % heat activated serum) showed the agglomeration state of the ENMs and changes in surface charges of cationic and anionic Ag ENMs.

### Uptake and subcellular localization of Ag ENMs of different charge and surface compositions

**Fig. 2:**
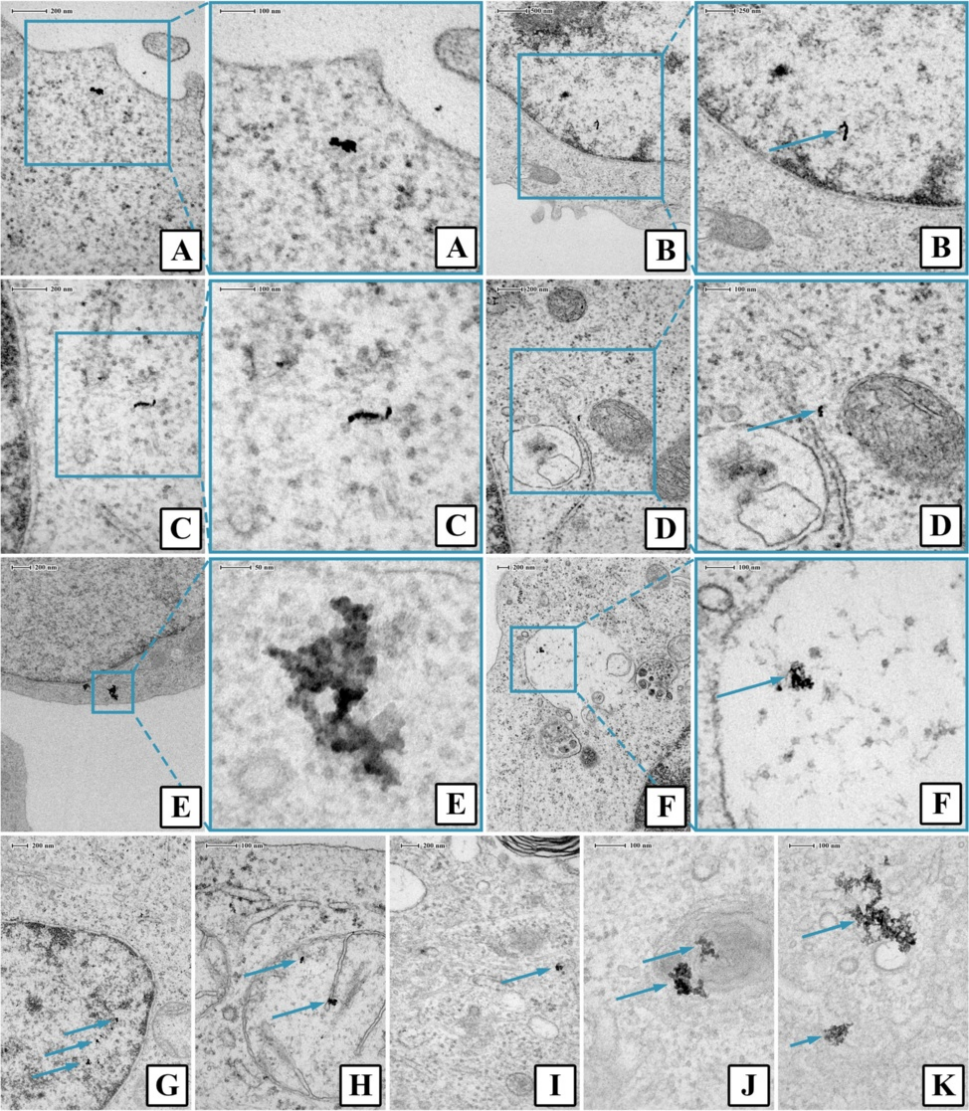
Cellular localisation of different Ag ENMs in TK6 cells after 24 h exposure. **a** Ag_Byk in cytoplasm; (**b**) Ag_Byk in nucleus; (**c**) Ag_Citrate in cytoplasm; (**d**) Ag_Citrate in cytoplasm; (**e**) Ag_SDS in cytoplasm; (**f**) Ag_Chitosan in vacuoles; (**g**) Ag_Chitosan in nucleus; (**h**) Ag_Chitosan in mitochondrion; (**i**) Ag_Disperbyk in cytoplasm; (**j**) Ag_Disperbyk in membrane structure; (**k**) Ag_Tween in cytoplasm

The uptake and subcellular localization of the Ag ENMs were confirmed using TEM. First, the images confirmed that all tested Ag ENMs were taken up by TK6 cells after exposure duration of 24 h (Fig. 2). Positively charged ENMs, Ag_Byk and Ag_Chitosan were the only Ag ENMs found in nuclei (Fig. 2b, g). Ag_Chitosan was detected in nuclei, vacuoles and mitochondria, and was present mostly as large Ag ENM agglomerates (Fig. 2f, g, h). Ag_Byk was present in nuclei and cytoplasm, as small Ag ENM clusters or single ENMs (Fig. 2a, b). Additionally we detected single, non-agglomerated Ag ENMs in organelles with membranes, probably deformed vacuoles (picture not shown). Ag ENMs, with negative and neutral charge were not observed to interact directly with mitochondria or nuclei in any of the images assessed. Agglomerates of Ag_SDS, Ag_Tween and Ag_Citrate were detected in cytoplasm (Fig. 2c, d, e, k). Ag_Disperbyk was located in vesicles, very close to the cell surface or in cytoplasm (Fig. 2j). The extent of Ag ENM intracellular localization could not be fully evaluated, due to their high toxicity. In tested concentrations many cells in stages of necrosis and apoptosis were observed. Due to cytoplasm condensation, detection of Ag ENMs was difficult. It is possible that small (not agglomerated) ENMs bypassed detection.

### Impact of Ag ENM charge and surface composition on cell proliferation and cell membrane damage

**Fig. 3:**
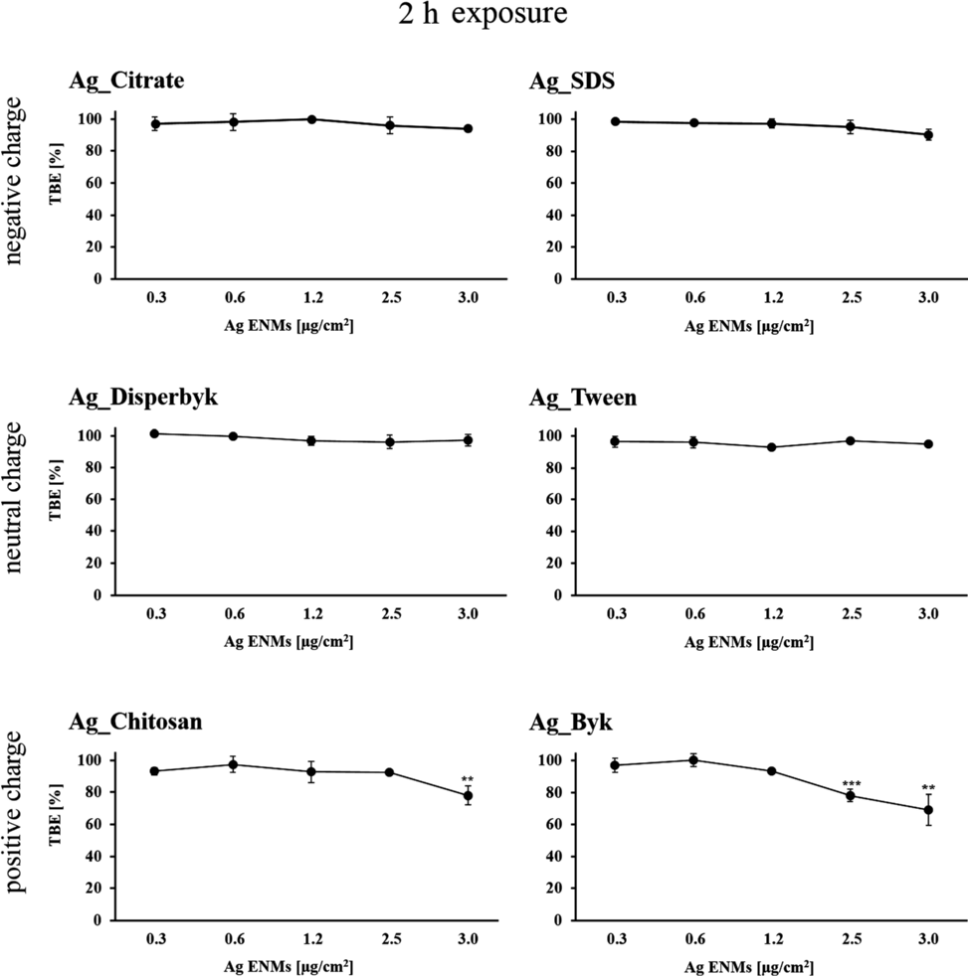
Cytotoxic and cytostatic effects of Ag ENMs with different charge (negative: Ag_Citrate and Ag_SDS; neutral: Ag_Disperbyk and Ag_Tween; positive: Ag_Chitosan and Ag_Byk) on TK6 cells measured as trypan blue exclusion (TBE) Cells were treated with 5 concentrations (μg/cm^2^) of Ag ENMs for 2 h and cell number was counted immediately after the exposure. Graphs represent cytotoxicity relative to 100 % of control. The data are expressed as mean ± SD of three independent experiments. *P* values indicate statistically significant results; *p < 0.05; **p < 0.01; ***p < 0.001

**Fig. 4:**
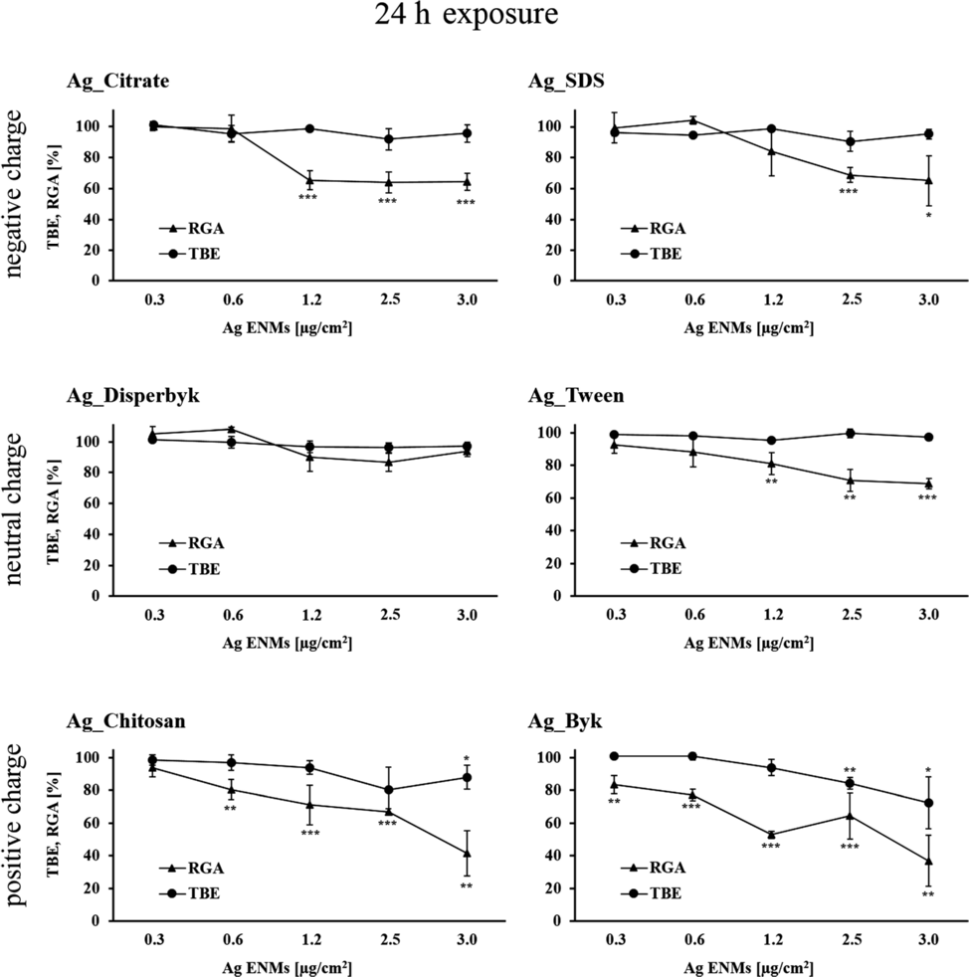
Cytotoxic and cytostatic effects of Ag ENMs with different charge (negative: Ag_Citrate and Ag_SDS; neutral: Ag_Disperbyk and Ag_Tween; positive: Ag_Chitosan and Ag_Byk) on TK6 cells measured as trypan blue exclusion (TBE) and Relative growth activity (RGA). Cells were treated with 5 concentrations (μg/cm^2^) of Ag ENMs for 24 h and cell number was counted immediately after the exposure. Graphs represent cytotoxicity relative to 100 % of control. The data are expressed as mean ± SD of three independent experiments. *P* values indicate statistically significant results; *p < 0.05; **p < 0.01; ***p < 0.001

The effect of Ag ENMs with different charge (negative: Ag_Citrate and Ag_SDS; neutral: Ag_Disperbyk and Ag_Tween; positive: Ag_Chitosan and Ag_Byk) on membrane damage and proliferation of TK6 cells was examined after 2 and 24 h exposure using TBE and RGA. Ag ENM stabilizers were used as controls and the concentration of stabilizer corresponds to the concentration of stabilizer in the highest concentration of Ag ENMs. Data are presented relative to the control cells that had no Ag ENM treatment (negative control). A clear concentration response in RGA was observed for almost all tested ENMs (except Ag_Disperbyk). Positively charged Ag ENMs, Ag_Chitosan and Ag_Byk were considered the most cytostatic of all tested materials (Figs. 3 and 4). Also only positively charged Ag ENMs caused cell membrane damage, already evident during the 2 h exposure. Of the tested Ag ENM stabilizers, none had toxic effects at tested concentration (data not shown).

### Impact of Ag ENM charge and surface composition on cell morphology

**Fig. 5:**
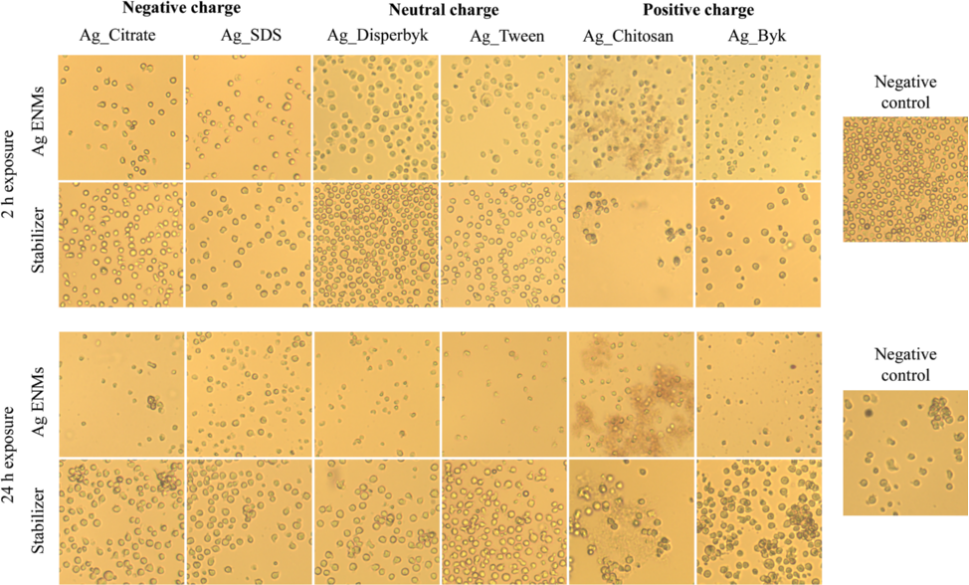
Light microscopy (100×) of TK6 cells incubated with Ag ENMs with different charge and surface composition (negative: Ag_Citrate and Ag_SDS; neutral: Ag_Disperbyk and Ag_Tween; positive: Ag_Chitosan and Ag_Byk). Cell were treated with Ag ENMs, concentration 2.5 μg/cm^2^ or equal concentration of Ag ENMs stabilizers for 2 or 24 h, and pictures were taken immediately after exposure

Morphological changes of TK6 cells exposed to Ag ENMs with different charge and surface coating were observed using an optical microscope (Fig. 5). After 2 h exposure, changes of cellular morphology were observed in cells treated with Ag Byk ENMs compared to untreated cells. After short exposure cells were shrunken and irregular in shape. In samples with Ag Chitosan, clumps of cells were observed. However, the same changes were seen in cells treated with chitosan without Ag ENMs. After 24 h exposure, changes in cell morphology, such as shrunken cells with irregular shape, were observed in all samples. After 24 h, fewer or no colonies were observed, which strongly suggests that Ag ENMs had an impact on cell proliferation. No morphology changes were observed in stabilizer controls except for cells treated with chitosan, where characteristic “protein structures” were observed. This could be explained by precipitation of chitosan in biological media at pH ~ 7 or by interaction of chitosan with protein.

### Impact of Ag ENM charge and surface composition on level of DNA strand breaks

**Fig. 6:**
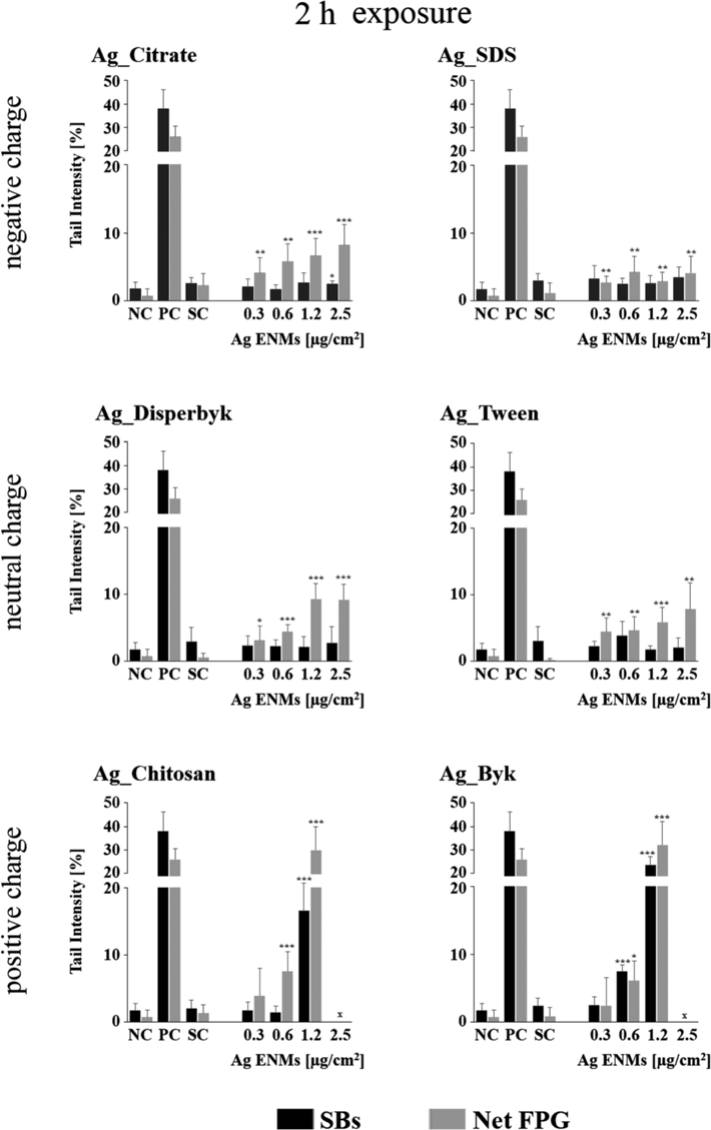
Effects of surface charge and surface coating on Ag ENMs genotoxicity in TK6 cells. DNA damage (strand breaks and oxidised DNA lesions expressed as Net FPG) measured by the Comet assay in TK6 cells exposed to six different Ag ENMs for 2 h Data are presented as mean values ± SD. *P* values indicate statistically significant results; *p < 0.05; **p < 0.01; ***p < 0.001. NC – negative control; PC – positive control (strand breaks: H_2_O_2:_ 50 μM, 5 min, in ice, Net FPG: Ro19-8022: 1 μM, plus visible light, 5 min, in ice); SC – stabilizer control (concentration of stabilizer is equivalent to the concentration of stabilizer in Ag ENMs 2.5 μg/cm^2^). x –level of DNA damage could not be fully evaluated, due to strong cytotoxicity

**Fig. 7:**
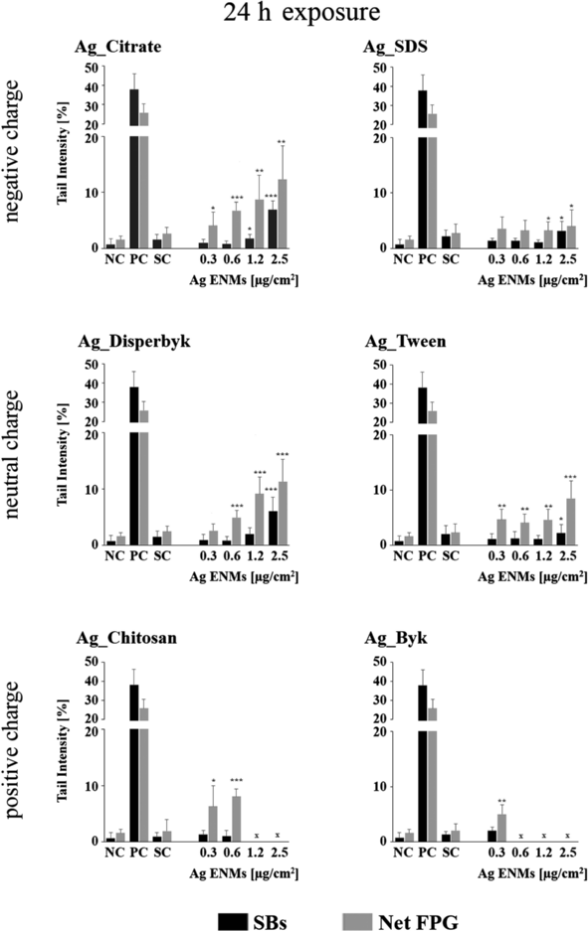
Effects of surface charge and surface coating on Ag ENMs genotoxicity in TK6 cells. DNA damage (strand breaks and oxidised DNA lesions expressed as Net FPG) measured by the Comet assay in TK6 cells exposed to six different Ag ENMs for 24 h. Data are presented as mean values ± SD. *P* values indicate statistically significant results; *p < 0.05; **p < 0.01; ***p < 0.001. NC – negative control; PC – positive control (strand breaks: H_2_O_2:_ 50 μM, 5 min, in ice, Net FPG: Ro19-8022: 1 μM, plus visible light, 5 min, in ice); SC – stabilizer control (concentration of stabilizer equivalent to concentration of stabilizer in Ag ENMs 2.5 μg/cm^2^). x – level of DNA damage could not be fully evaluated, due to strong cytotoxicity

The standard alkaline CA was employed for detection of single- and double-strand breaks in TK6 cells exposed to Ag ENMs with 3 different charges (negative: Ag_Citrate and Ag_SDS; neutral: Ag_Disperbyk and Ag_Tween; positive: Ag_Chitosan and Ag_Byk) (Figs. 6 and 7). Two different time points were investigated; 2 and 24 h. Ag ENM stabilizers were used as controls. The strongest effects were observed for positively charged Ag ENMs, already after 2 h exposure. High levels of strand breaks were also present at the highest tested concentration (2.5 μg/cm^2^) but could not be evaluated due to software limitation (DNA damage: >70 % tail intensity). However, Ag_Chitosan and Ag_Byk were also found to induce toxic effects (RGA 60–80 %) (Fig. 7). Slight but significant increases in DNA breaks were found also in cells exposed to Ag_Citrate. Neutral charge Ag ENMs, and also Ag ENMs coated with SDS, did not cause DNA damage after short exposure. We found that after long-term exposure all tested Ag ENMs induce DNA damage. However the level of DNA damage could not be fully evaluated for cationic ENMs as comets/cells were not detected under the microscope. Ag_Citrate and Ag_Disperbyk increased DNA damage at a concentration of 1.2 μg/cm^2^ but statistically significant differences were not found. No increases in DNA damage were seen for any of the tested Ag ENM stabilizers at either time point. Summing up, we conclude that the impact of Ag ENMs on the level of strand breaks is both charge- and surface coating-dependent.

### Impact of Ag ENM charge and surface composition on level of DNA oxidation

The modified CA was employed for detection of DNA oxidation in TK6 cells exposed to Ag ENMs with different charges following the same conditions as mentioned above (Figs. 6 and 7). Results indicated that almost all tested Ag ENMs induced DNA oxidation and the level of DNA oxidation was similar to the level of strand breaks.

Among all the tested materials, Ag ENMs stabilized by SDS were found to be the least genotoxic; only a slight (in the range of background level), but statistically significant, increase in DNA oxidation was detected.

### Impact of Ag ENM charge and surface composition on gene mutation in V79-4 cells

**Fig. 8:**
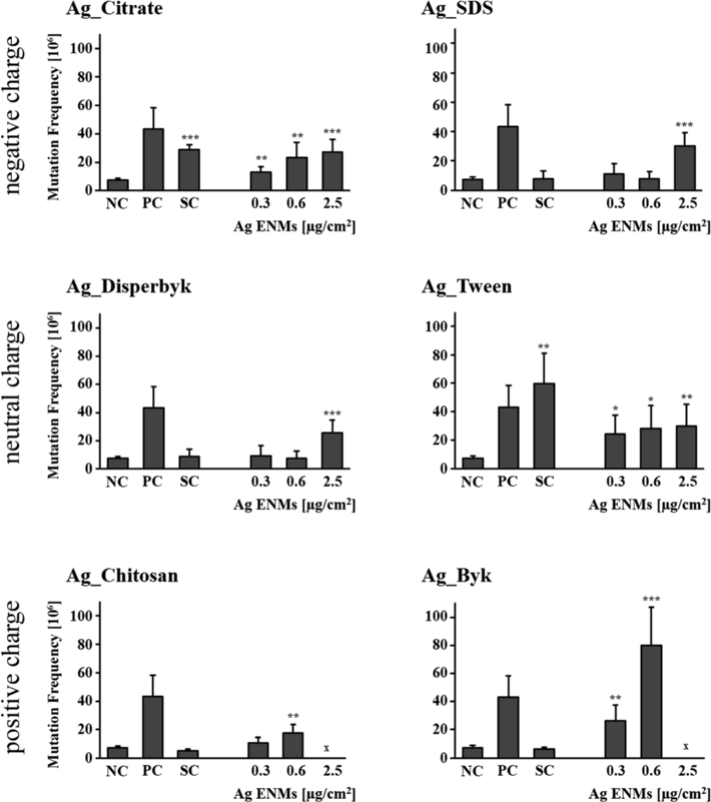
Effects of surface charge and surface coating on Ag ENMs on induction of *HPRT* gene mutations in V79-4 cells. Cells were treated with 3 different concentrations of Ag ENMs and Ag ENM stabilizer equivalent to stabilizer concentrations in highest tested concentrations of Ag ENMs. The mutant frequencies (×10^6^) are expressed as mean ± SD of three independent experiments, with two independent harvests per experiment. MMS (0.03 μM, 30 min) was used as a positive control. NC – negative control; PC – positive control (MMS; 0.03 μM, 30 min), SC – stabilizer control . For Ag_Byk and Ag_Chitosan level of mutants could not be fully evaluated, due to strong cytotoxicity. Significant difference from unexposed control (*p < 0.05, **p < 0.01, ***p < 0,001)

**Fig. 9:**
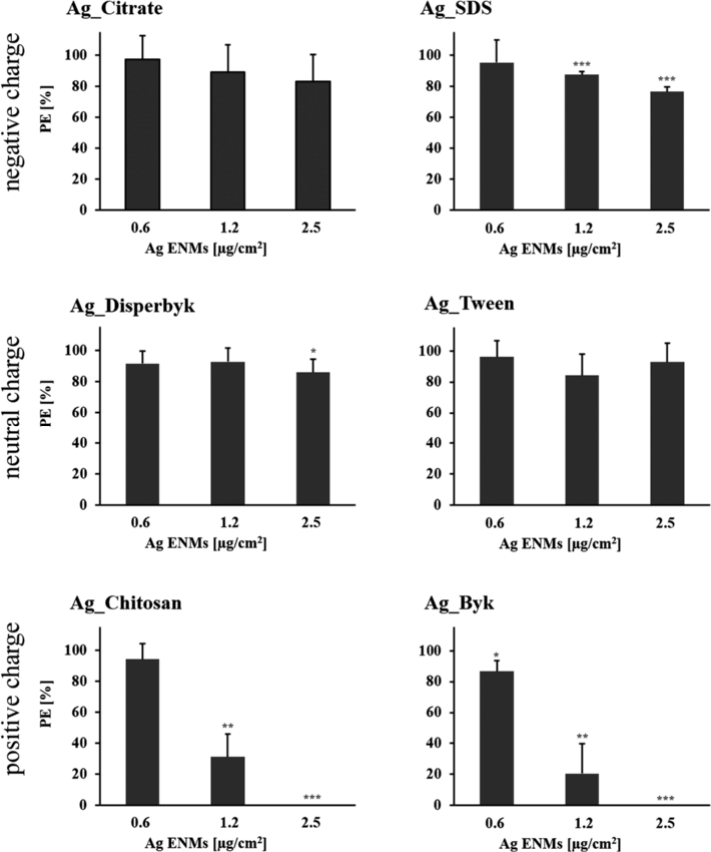
Cytotoxicity effect measured by Plating efficiency (PE) assay. Bars represent cytotoxicity relative to 100 % of untreated cells, expressed as mean ± SD of three independent experiments. The data are expressed as mean ± SD of three independent experiments. *P* values indicate statistically significant results; *p < 0.05; **p < 0.01; ***p < 0.001. Cytotoxic effect was not observed for any of stabilizers tested (data not shown)

The mutagenic potential of Ag ENMs with different charges was examined by the *HPRT* gene mutation assay according to OECD guideline 476 (Fig. 8). Three independent experiments, each with two mutant harvests, were performed with V79-4 cells. Cytotoxic effects were investigated in parallel with the PE assay (Fig. 9). Ag ENM stabilizers were used as controls. All tested materials induced *HPRT* gene mutations in V79-4 cells; however, the mutagenic potential of Ag ENMs is not charge- but only surface coating-dependent. The highest level of mutants was found in cells exposed to Ag_Byk which also showed highest cytotoxicity (measured by PE). Of six tested Ag ENM stabilizers, sodium citrate and Tween have a strong impact on induction of gene mutants. The level of mutants in cells treated with Ag ENMs stabilized by citrate is equal to the level of mutants found in the stabilizer control. Sodium citrate and Tween showed no cytotoxicity (PE assay).

### Release of Ag ions from Ag ENM in a biological medium

Amounts of dissolved Ag ions after 24 h incubation in protein-supplemented cell culture medium were evaluated by ICP-MS (Table 3). We found that Ag ENM surface charge and chemical composition had no impact on Ag ENM dissolution rate. Relatively similar concentrations of released Ag ions were observed in all tested samples.

**Table 3:** Concentrations of Ag ions in Ag ENM samples and Ag ions released after incubation in cell culture media were evaluated by ICP-MS. Ag ions in Ag ENM stock were expressed as % of total Ag in Ag ENM samples. Ag ions were isolated from Ag ENMs by (a) extraction and (b) by ultracentrifugation. Ag ions released during 24 h incubation in cell culture medium were quantified by CP-MS and expressed as percentage of the total added Ag ENMs. Results are presented as mean ± standard deviation of 3–6 replicates

	Ag ions (%) in stock		Released Ag ions after 24 h incubation in cell culture medium
	Obtained by extraction	Obtained by ultracentrifugation	
Ag_Citrate	12.16 ± 5.19	8.41 ± 0.64	7.02 ± 3.27
Ag_SDS	13.81 ± 2.49	7.28 ± 2.56	6.44 ± 1.93
Ag_Disperbyk	25.72 ± 4.33	15.86 ± 0.49	5.25 ± 1.37
Ag_Tween	19.12 ± 1.93	7.72 ± 1.45	6.68 ± 1.87
Ag_Chitosan	x	14.37 ± 9.6	11.34 ± 6.37
Ag_Byk	x	13.59 ± 5.63	3.94 ± 0.04

x – Ag ion concentrations cannot be determined for the positively charged Ag ENMs using this approach

## Discussion

Risk assessment strategies for ENM testing require relevant *in vitro* toxicology data, based on well-designed experiments. *In vitro* tests can be successfully applied in nanotoxicology studies. However, reference and quality standards should always be included: materials with well-defined physico-chemical properties, range of controls (including stabilizer controls) and representative cell models are crucially important. Also physical processes such as dissolution, aggregation and sedimentation should be taken into consideration to better understand the mechanism of ENM toxicity. The main reason for our study was to investigate the impact of surface charge and chemical composition of Ag ENMs on viability, genotoxicity and mutagenicity. As the most common cell model for genotoxicity and mutagenicity studies, V79-4 and TK6 cells were used. Additionally, ENM aggregation and dissolution in cell culture medium, as well as uptake and subcellular localization were investigated as crucial aspects of ENM toxicity. Data from all experiments are summarized in Table 4.

**Table 4:** Summary of cellular responses to Ag ENMs with different surface charge and chemical composition

Surface charge:			Negative		Neutral		Positive	
Surface chemical composition:			Citrate	SDS	Disperbyk	Tween	Chitosan	Byk
Intercellular localisation	Cytoplasm	24 h	+	+	+	+	+	+
	Nucleus	24 h	-	-	-	-	+	+
	Mitochondria	24 h	-	-	-	-	+	-
Cytotoxicity	Cells morphology	2 h	-	-	-	-	+ (s)	+
		24 h	+	+	-	-	+ (s)	+
	Trypan blue exclusion	2 h	-	-	-	-	+	++
		24 h	-	-	-	-	++	++
	Relative Growth Activity	24 h	++	++	-	++	+++	+++
Genotoxicity and mutagenicity	Comet assay – Strand breaks	2 h	+				++	++
		24 h	++	+	++	+	(x)	(x)
	Comet assay – Oxidative DNA lesions	2 h	+++	+++	+++	+++	+++	+++
		24 h	+++	++	++	+++	(x)	(x)
	*HPRT* gene mutation	24 h	++ (s)	+	+	++ (s)	+	++

The relative tendency of each Ag ENMs to induce cellular responses related to uptake, viability, cytotoxicity, genotoxicity and mutagenicity after 2–24 h exposure

(−) no harmful effects were observed in any of tested concentrations

(+) indicates a significant increase in the intracellular event in one from three-five tested concentrations/localisation in selected cells compartments/change in cells morphology

(++) indicates a significant increase in the intracellular event in two-three from three-five tested concentrations

(+++) a significant increase in the intracellular event in one from four-five tested concentrations

(s)- Toxic effect of Ag ENMs stabilizers

(x)- Fully biological effect could not be evaluated due to strong method saturation

In the last decade, the toxicity of Ag ENMs has been widely investigated, using different model systems: stem cells or cell lines, bacteria, higher plants and fungi [1, 5, 7, 8, 14–21, 34]. Comparison of nanotoxicology results between studies and research approaches is a challenge, mostly because of variance in tested materials and differences in conditions of experiments. Most researchers in nanotoxicology express ENM concentrations in mass units, usually μg/ml. From our previous work, we conclude that when comparing materials with different size and surface area, it is more useful and realistic to express concentrations as number of ENMs or as surface area of ENMs, than as mass units [25]. ENMs can change during exposure, mostly due to reactions with biomolecules present in the medium, for example proteins binding to the surface of ENMs, affecting their aggregation and sedimentation [35]. For this reason it is crucial to keep a constant number of ENMs per surface area of exposed cells in each experiment, depending on exposure conditions (such as size of plate or medium volume). Each laboratory has its own validated protocols, including cell density or medium composition. These parameters can also have significant impact on final results and can cause difficulties in comparisons with other research groups [13, 35]. In our study, we optimized exposure volume, to keep the same amount of ENMs per surface area in all experiments. We also expressed concentrations as ENMs per cell [μg/cell and ENM cm^2^/cell] to simplify comparison with studies of other researchers, according to the proposals of Stone *et al.* and Huk *et al.* [13, 36]. Nanotoxicology research requires quality assurance, with the inclusion of a range of controls to exclude false positive/negative results, not related to nano-responses. In the *HPRT* gene mutation assay, we observed strong mutagenic effects with two of six tested stabilizers: sodium citrate and Tween 80. Toxicity of citrate-stabilized Ag ENMs was investigated previously [23, 37–39]. However, in none of these studies was the toxicity of stabilizers taken into consideration. In our study, we found a strong mutagenic effect of sodium citrate at a concentration 0.002 % w/v and of Tween 80 at a concentration of 0.008 % w/v. Similar findings were already reported by Wang *et al.*, who have shown that photo-mutagenic effects of gold ENMs are related not only to ENMs but also to citrate ions used as a stabilizer [40]. Our results have an implication for the ‘safety by design’ approach, indicating that stabilizers with mutagenic potency should not be used in ENM synthesis.

Additionally, we observed unusual phenomena in samples treated by chitosan, under the light microscope. Abnormal clumps were present, in both cells treated by Ag_Chitosan and cells treated only with chitosan. Also in DLS characterization of Ag_Chitosan in biological medium, Ag ENM agglomerates were about 10 times bigger than found with other Ag ENMs. Precipitation of chitosan at the neutral pH of cell culture medium is a possible explanation of this observation.

The use of well-characterized reference ENMs is a crucial aspect of *in vitro* nanotoxicology studies, because the toxic potential of ENMs is strictly dependent on their physico-chemical properties. Hydrodynamic size plays a crucial role in the interaction of ENMs with living organisms. Smaller Ag ENMs were found to be more cytotoxic, genotoxic and stronger inducers of ROS production compared to bigger Ag ENMs [23–27, 41]. Size-dependent toxicity of Ag ENMs is combined with faster kinetics of dissolution and higher numbers and surface area of ENMs [23–25]. Shape is the next parameter shown to have a significant impact on Ag ENM toxicity. It was found that whereas Ag ENMs strongly affected cell viability and cytotoxicity and decreased the calcium level in epithelial lung cells, spherical Ag ENMs had no harmful effect in the same concentration range [42]. In a bacterial model, triangular Ag ENMs had a bigger effect on *E. coli* compared with spherical and rod shaped ENMs [43]. Chemical composition of the ENM surface is another crucial characteristic. Citrate-stabilized Ag ENMs were found to have a stronger effect on human skin keratinocytes cytotoxicity than PVP-stabilized Ag ENMs [39]. In addition, much higher expression of Rad51 protein, a biomarker of double strand breaks, was found in mouse embryonic stem cells and mouse embryonic fibroblasts treated with polysaccharide surface functionalized Ag ENMs in comparison to non-functionalized Ag ENMs [44]. The last significant parameter which seriously affects Ag ENM toxicity is surface charge. It is believed that positively charged ENMs are potentially more harmful than materials with negative or neutral charge. Suresh *et al.* have shown that the cationic Ag ENMs stabilized by poly(diallyldimethylammonium) chloride had a stronger impact on cell membrane integrity and cytotoxicity compared with anionic Ag ENMs [28]. Higher DNA damage was also observed in human fibroblasts and fibrosarcoma cells exposed to cationic iron oxide magnetic ENMs, whereas at the same concentrations anionic ENMs showed only a slight or no effect [45].

Since toxicity of ENMs is strictly related to their physico-chemical properties, all studied materials were synthesized in such a way that the only parameter differentiating them was their surface chemistry. In addition, to distinguish between effects of surface charge and surface chemical composition two different stabilizers per charge were selected.

For our study, ENMs with different surface charge were selected. However, after 24 h incubation of Ag ENMs in cell culture medium, we observed changes in zeta potential of cationic and anionic ENMs. This phenomenon is related to the binding of biomolecules onto the surface of ENMs, a process named protein corona formation. Due to high surface area, ENMs progressively and selectively adsorb biomolecules, such as proteins and lipids, when they come into contact with complex biological fluids [46, 47]. Formation of a protein corona is a dynamic process, because different molecules are attracted to the ENM surface at different time points. Schlinkert *et al.* [48] demonstrated that cationic Ag ENMs changed their surface charge, depending on how long they are incubated in cell culture medium. What is more, the chemical composition of the protein corona is also dependent on ENM surface charge, as different molecules will be attracted by cationic and anionic ENMs [49]. Cationic ENMs decrease zeta potential, in contrast to anionic ENMs. We have not observed significant changes in zeta potential of neutral ENMs, consistent with previous reports, which can be explained by low interaction between neutral ENMs and proteins [49].

The cytotoxic effect of Ag ENMs has been widely studied, and many researchers have reported that Ag ENMs could reduce cell viability, cause cell membrane damage, decrease the level of calcium, cause perturbation of mitochondria, inhibit cell proliferation, or induce apoptosis and necrosis [14–21]. In our study we found that cationic Ag ENMs are the most cytotoxic; they strongly affected cell membrane and cell morphology, inhibited proliferation and induced cells death, consistent with previous reports [28].

Genotoxicity testing in a regulatory perspective requires a battery of tests addressing these different genotoxic and mutagenic endpoints, since no single method is capable of detecting all different forms of genome damage including DNA lesions, chromosome aberration or mutation [50, 51].

Our results showed that most of the tested Ag ENMs were genotoxic, and significantly increased the levels of oxidized purines and DNA strand breaks, consistent with our previous experiments and other reports [15, 23, 25, 52]. The most significant effects were observed in cells treated with the two cationic Ag ENMs, Ag_Byk and Ag_Chitosan, with significant genotoxic effects already after 2 h exposure. However, cytotoxicity was often seen, especially after 24 h exposure. Early apoptosis or necrosis may lead to an elevated % DNA in the tail giving false positive results in the CA. However, in our case, maximum cytotoxicity was 20–30 %, at the highest concentration used, and so a significant influence on the CA results is unlikely.

To investigate the impact of Ag ENMs and surface composition on mutant frequency, we applied the *HPRT* gene mutation assay. Only a few studies of mutagenic effects of Ag ENMs are available so far [53–56]; most employed the Ames test, an assay which has strong limitations when applied in nanotoxicology [50]. A study using a mammalian model also reported no mutagenic effects of Ag ENMs; however, it is difficult to refer to this study because of big differences in the materials used, and lack of detailed characterization [56].

In our study, to investigate the impact of Ag ENMs on gene mutation, instead of the Ames test, the X chromosome-linked hemizygous *HPRT* gene mutation assay was applied. The main advantages of this assay are that it is based on a mammalian cell line, selection of mutants with 6-thioguanine is simple, there is no interference with nanomaterials, it has the capacity to characterise the diverse range of possible mutations, and only one allele needs to be inactivated for the mutation to be expressed [57].

In our study we found that all six tested Ag ENMs induced mutants in V79-4 cells. We have to exclude false positive results of Ag_Tween and Ag_Citrate, due to strong mutagenic effects of Ag ENM stabilizers. Among all tested Ag ENMs Ag_Byk showed a clear positive effect and could be considered as mutagenic. Ag_SDS, Ag_Disperbyk and Ag_Chitosan were found to be weakly positive and further investigation would be needed to confirm their mutagenicity. In our previous experiments on nanomaterial mutagenicity, Ag ENMs with sizes 50, 80 and 200 nm were tested [25]. In that study, we observed a clear reverse concentration-response, for all tested materials. Discrepancies between these two studies can be explained by differences in investigated materials. In a study of size-related toxicity, PVP was applied to prevent protein binding to the surface of Ag ENMs, Ag ENM agglomeration and dissolution [49, 58]. PVP-stabilized Ag ENMs keep their primary shape and size during all exposure times in contrast to Ag ENMs tested in the present experiment. Different ranges of tested concentrations can also explain other biological responses. In a study of size-related toxicity, a wider concentration range of Ag ENMs was used (0.21–15.9 μg/cm^2^) [25]. At a high concentration, there was a dramatic increase and accumulation of various kinds of DNA damage, and mutants lost their survival ability, which can explain the reverse-concentration trend. We also observed that the mutagenic potential of Ag ENMs is dependent to some degree on cytotoxicity. Ag ENMs with negative and neutral charge (also PVP-stabilized Ag ENMs) decreased cell viability only slightly or not at all, but greatly induced the frequency of *HPRT* gene mutants in contrast to Ag_Byk and Ag_Chitosan, which are highly cytotoxic (even after washing) and also greatly induced gene mutations in V79-4 cells. In summary Ag ENMs can cause gene mutations, at both non-cytotoxic and cytotoxic concentrations, depending on their physico-chemical properties. Low concentrations of Ag ENMs increased the level of *HPRT* gene mutants, up to the point at which the level of DNA damage was so high that mutants lost their survival ability.

High cytotoxic and genotoxic effects of Ag_Chitosan and Ag_Byk can be explained by different uptake mechanisms and subcellular localization of ENMs. The cell membrane, due to its negative charge, is more attractive for cationic ENMs rather than anionic. Kralj *et al.* [59] in their study on charge dependent uptake, showed that positively charged, magnetic ENMs were internalized by a human breast epithelial cell to a much greater extent than were negatively charged ENMs. However, ENM charge did not have any impact on subcellular localization, because both cationic and anionic magnetic ENMs were localized in lysosomes. Moreover Asati *et al.* [60] found that cerium oxide ENMs with neutral charge were found mostly in cytoplasm, in contrast to cationic and anionic ENMs, which were located in both cytoplasm and lysosomes.

In our study, we found that all six tested Ag ENMs were taken up by TK6 cells, and Ag ENMs were present mostly in the cytoplasm. However, we also found cationic ENMs in the nucleus and mitochondria. The presence of ENMs in these organelles was already reported [15, 16, 25, 61] and suggests that Ag ENMs can have direct contact with DNA. Ag ENMs can enter the nucleus by penetration or during mitosis and interact or bind with DNA molecules or affect DNA replication and transcription [62], which could explain the much higher level of different DNA lesions in cells treated by cationic Ag ENMs compared with anionic or neutral.

During exposure in cell culture medium, Ag ENMs can slowly dissolve to Ag ions. Ag ions can reduce cell viability and stimulate ROS production; however, it is still not clear how significant an impact the Ag ions have on total Ag ENM toxicity [17, 63]. The experiments with an ion fraction (obtained from Ag ENMs by ultracentrifugation after incubation of Ag ENMs in cell culture medium), have shown no effect on cell viability or genotoxicity [23, 27, 42, 58]. This is mostly because the concentrations of released ions were relatively low, at 2–20 % of the applied concentration of Ag ENMs (depended on Ag ENMs shape, size and surface coating), and were found to be insufficient to cause toxic effects. A significant fact is also that Ag in nano-form is more efficiently taken up by the cells than is Ag in ion form [64, 65].

In our study we found no significant impact of surface composition/charge on the dissolution rate of Ag ENMs in cell culture medium, consistent with a previous report [23]. Our results suggested that subcellular Ag ENM dissolution, rather than extracellular, plays significant roles in Ag ENM toxicity.

In our experiments, both cationic Ag ENMs caused membrane damage, inhibited cell proliferation and induced strand breaks and DNA oxidation. However, Ag_Byk were also found to be mutagenic and induced cell cytotoxicity at lower concentration than Ag_Chitosan. We found that Ag_Byk created much smaller agglomerates in the rich protein medium (~40 nm) compared to Ag_Chitosan (~700–1200 nm), which suggests that not only dissolution of Ag ions, but also agglomeration state can have a crucial impact on Ag ENMs toxicity, as already suggested by Lankoff *et al.* [23] and Gliga *et al.* [61]. Agglomerated Ag ENMs were found be less toxic. Several authors suggest that it may be mostly due to reduction of specific surface area, slowing the ENM dissolution, and inhibition of ENM uptake [23, 30, 61].

## Conclusion

We investigated the impact of Ag ENM surface charge and composition on cell cytotoxicity, genotoxicity and mutagenicity. Agglomeration, dissolution and uptake were additionally investigated as crucial aspects of Ag ENM toxicity. We found that positive Ag ENMs had greater impact on cell proliferation, cell death, membrane disintegration and DNA damage than Ag ENMs with neutral or negative charge. Severe genotoxic effects of cationic Ag ENMs can be combined with the presence of Ag ENMs in the nucleus and mitochondria, which suggests that Ag ENMs can induce toxicity by both direct contact with DNA and indirect (via oxidative stress) mechanisms. Our experiments with the *HPRT* gene mutation assay demonstrated that not only surface charge but also surface chemical composition play a significant role in Ag ENM toxicity.

## Abbreviations

CA: Comet assay
DLS: Dynamic light scattering
ENMs: Engineered nanomaterials
FPG: Formamidopyrymidine DNA glycosylase
OECD: Organization for Economic Co-operation and Development
PE: Plating efficiency
PVP: Polyvinylpyrrolidone
RGA: Relative growth activity
SDS: Sodium dodecyl sulphate
TBE: Trypan blue exclusion assay
TK6: Human B-lymphoblastoid cell line
TEM: Transmission electron microscopy
V79-4: Chinese hamster lung fibroblast cells
XPS: X-ray photoelectron spectroscopy
XRD: X-ray diffraction
